# Enhanced renal ischemia/reperfusion injury repair potential of exosomes derived from B7-H1^high^ mesenchymal stem cells

**DOI:** 10.3389/fgene.2025.1516626

**Published:** 2025-04-02

**Authors:** Jiahui He, Yawei Yao, Ruiyan Wang, Yujia Liu, Xingyu Wan, Hao Wang, Yuqiang Zhou, Wenjing Wang, Yan Ma, Xinghua Lv

**Affiliations:** ^1^ Department of Anaesthesia, The First Clinical Medical College of Lanzhou University, Lanzhou, China; ^2^ Department of Day Surgery Center, The First Hospital of Lanzhou University, Lanzhou, Gansu, China

**Keywords:** renal ischemia-reperfusion injury, exosomes, B7-H1, cell sorting, C3, NF-κB

## Abstract

Two subgroups with high expression of B7-H1 and low expression of B7-H1 were successfully isolated from primitive human umbilical cord mesenchymal stem cells. And exosomes with high B7-H1 expression and low B7-H1 expression were successfully isolated. In comparison to the sham-operated group, mice in the IRI group demonstrated elevated serum levels of blood urea nitrogen (BUN) and serum creatinine (Scr), accompanied by a more pronounced degree of renal tissue damage. The administration of exosomes via the tail vein markedly accelerated the recovery of renal function in IRI mice, with the therapeutic effect beingmore pronounced in those treated with B7-H1high-Exo. Moreover RNA sequencing of mouse kidney treated with B7-H1high-Exo and B7-H1low-Exo showed that eight genes (C3, IRF7, AREG, CXCL10, Aldh1l2, Fnip2, Vcam1, St6galnac3) were involved in the pathophysiological process of ischemia-reperfusion injury. The *in vitro* and *in vivo* experiments showed that the expression level of C3 protein was significantly decreased, which indicated that B7-H1high-Exo played a therapeutic role by down-regulating C3.

## Introduction

The occurrence of acute kidney injury (AKI) following cardiac surgery can reach up to 40% among patients undergoing such procedures. Even mild and transient forms of AKI are associated with an increased risk of prolonged intensive care unit (ICU) stay, as well as elevated morbidity and mortality rates (Lassnigg et al., 2004; Wang and Bellomo, 2017). Renal ischemia-reperfusion injury (IRI) represents the predominant etiology of perioperative AKI and has emerged as a critical determinant of patient prognosis. In a variety of clinical settings, including major vascular, cardiac, and hepatic surgeries, as well as conditions such as shock, sepsis, trauma, and renal transplantation, renal IRI arises from the cessation of renal blood flow, followed by reperfusion. This process initiates a signaling cascade that mediates necrosis, apoptosis, and inflammation of renal cells, leading to AKI, which is characterized by the activation of endothelial cells, leukocyte recruitment and infiltration, and death of tubular epithelial cells (Bonventre and Weinberg, 2003; Han and Lee, 2019).

Despite the extensive knowledge and depth of existing studies on IRI, there remains no clear consensus regarding the precise role of immune system dysfunction in hypoxia-induced multiorgan injury. Programmed cell death protein 1 (PD-1) and its primary endogenous ligand, programmed death ligand 1 (PD-L1), which is also known as B7-H1 or CD274, are pivotal immune checkpoint molecules that play a critical role in regulating apoptosis. B7-H1, part of the B7 family of co-stimulatory molecules, is an important target for immune regulation (Ishida et al., 1992). Recent research has shown that changes in B7-H1 expression are linked to IRI, with increased levels observed in various hypoxia experimental models and in patients experiencing IRI (Hakroush et al., 2021; Sumiyoshi et al., 2021; Wang et al., 2022). As early as 2003, Fondevila et al. suggested that liver damage from prolonged ischemia followed by reperfusion should be viewed as an inflammatory response driven by the innate immune system (Fondevila et al., 2003). They later provided initial evidence that the co-stimulation of PD-1-negative T-cells influenced a local innate immune-driven inflammatory response, leading to hepatic IRI. Indeed, while disruption of PD-1 signaling exacerbated hepatocyte injury, the engagement of B7-H1 following intentional stimulation conferred protection against fulminant IRI through a localized IL-10-mediated mechanism. These findings indicate that the engagement of negative PD-1/B7-H1 signaling is essential for maintaining liver homeostasis during IR-induced hepatocyte injury (Ji et al., 2010). While the numbers of studies in this domain is still limited, B7-H1 has demonstrated considerable potential as a clinical therapeutic target.

As novel mediators of intercellular communication, exosomes (Exo) play a pivotal role in stem cell-mediated tissue repair. Direct interaction with target cells enables the delivery of genetic materials, including mRNA and miRNA, bypassing the potential risks associated with stem cell transplantation (Gho and Lee, 2017; Guo et al., 2021).

Mesenchymal stem cells (MSCs) can be sourced from a variety of tissues, with hucMSCs being particularly advantageous for cell therapy due to their readily accessible origin from umbilical cords (Xiao et al., 2022). Despite the registration of approximately 300 clinical trials investigating the therapeutic potential of hucMSCs, their efficacy remains constrained by the inherent heterogeneity of MSCs and the adverse effects reported in some clinical trials (Phinney, 2012; Tyndall, 2011). Utilizing different MSCs can reduce negative effects, remove confounding factors, and improve the effectiveness of their specific roles, which in turn aids in creating more effective treatment options. Consequently, it is essential to further categorize and characterize MSCs according to their functional diversity to support the creation of standardized MSC-based approaches for treating various diseases (Wu et al., 2020).

Recent research has elucidated that human gingival mesenchymal stem cells (GMSCs) can be categorized into B7-H1^high^ and B7-H1^low^ subpopulations, with the immunomodulatory function of GMSCs being significantly associated with B7-H1 signaling. In a murine model of type II collagen-induced arthritis (CIA), the increased expression of B7-H1 led to a significant reduction in the activity of inflammatory cells activity compared to the B7-H1^low^ subpopulation, thereby mitigating inflammation in the CIA model. The presence of a GMSC subpopulation with elevated B7-H1 expression could offer a distinctive and complementary approach for stem cell-based treatments targeting autoimmune and inflammatory diseases.

The findings of these studies suggest that a subset of MSCs with elevated B7-H1 expression may offer a promising avenue for the treatment of IRI. However, the precise function of the CD274+ hucMSCs subset, along with the assessment of its Exo origin in the management of renal IRI and its underlying molecular processes, remains uninvestigated. In this study, subpopulations of B7-H1^high^ and B7-H1^low^ hucMSCs were isolated using flow cytometry, and Exo was subsequently extracted for further analysis. Advanced experiments have identified ST6GalNac3 as a critical mediator of B7-H1^high^-Exo function, with the *in vitro* effects on HK-2 cells attributed to the elevated expression of B7-H1. Our research discloses a novel role for B7-H1^high^-Exo, suggesting its potential in promoting renal tissue repair and functional recovery, thereby opening up promising avenues for cell-free therapeutic strategies in the treatment of IRI.

## Materials and methods

### Mice

Six-to eight-week-old male SPF-grade C57BL/6 mice were procured from the Animal Experiment Center of Lanzhou University [License: SCXK (GAN) 2023-0003] and housed in an SPF-grade laboratory. The mice were free to ingest food and water throughout the duration of the experiment. Environmental conditions, including constant temperature and humidity, were rigorously maintained.

### Mouse renal I/R model

Mouse renal I/R model was performed in male C57BL/6 mice. Briefly, the mice were anesthetized with pentobarbital sodium by intraperitoneal injection and lay on the platform. B7-H1^high^-Exo (50 μg), B7-H1^low^-Exo (50 μg) and PBS was separately injected into the tail vein of the fixed mice using a mouse tail vein injection imager before the start of surgery. Dorsal incisions of both left and right sides were made to expose kidneys. The right kidney artery was gently separated with cotton swabs and occluded with a microvascular clamp to induce renal ischemia for 45 min. The left renal pedicle clamping and ischemia were the same as right. After ischemia, the micro-aneurysm clips were removed to start the reperfusion. The wounds were sutured and resuscitated with warm sterile saline intraperitoneally. All operations were the same in the sham group except for clamping and ischemia. The mice were sacrificed 24 h after reperfusion and the specimens were collected. All animal experimental protocols were performed according to the guidelines of the Ethical Committee of the First Hospital Lanzhou University.

### Cell culture

HucMSCs, provided by Yinfeng Biologicals and validated by the bioassay laboratory of Shaanxi Stem Cell Engineering Co., Ltd., were cultured in Dulbecco’s Modified Eagle Medium (DMEM) supplemented with 10% fetal bovine serum (FBS), 1% penicillin, and streptomycin, and subsequently digested with 0.25% trypsin. After 48 h, the supernatant of MSCs cells transfected with B7-H1^high^ and B7-H1^low^ were collected for exosomes extraction. All cell cultures were maintained in a saturated humidity incubator at 37°C with 5% CO_2_, with the culture medium being replaced daily.

### HK2 cell culture and H/R model

HK2 cells purchased from Procell were cultured in DMEM/Nutrient Mixture F12 supplemented with 10% FBS, 500 U/mL penicillin, and 500 μg/mL streptomycin (Gibco) at 37 °C in a humidified atmosphere containing 5% CO2. For H/R treatment, HK2 cells were exposed to hypoxia condition with 1% O2, 5% CO2, and 94% N2 for 24 h in the absence or presence of B7-H1^high^-Exo and B7-H1^low^-Exo (50 μg/mL). Then reoxygenation (21% O2, 5% CO2, and 74% N2) for 4 h. Samples were collected after modeling for analyses.

### MACSQuant®Tyto^®^ cell flow sorting and flow cytometry

hucMSCs were digested with 0.25% trypsin and subsequently incubated with anti-CD90-APC and anti-CD274-PE antibodies (Biolegend, United States) at 25°C for 30 min. The fluorescent cells that had been labeled with anti-CD90 antibody and anti-CD274 antibody were transferred to a MACSQuant^®^ Tyto^®^ sorting bin. The MACSQuant Tyto Running Buffer contained 1 × 10^7^ hucMSCs per 10 mL. Before sorting, logical gating hierarchies were established using MACSQuant Tyto software. Cell debris, doublets, and dead cells were excluded, and a gate was set to isolate the target cell population. Samples were sorted at a flow rate of 4 mL/h under a pressure of approximately 140 mbar. Upon completion of the sorting process, B7-H1 expression in both positive and negative cell populations was analyzed using a NovoCyte Advanteon Dx VBR flow cytometer.

### RT–qPCR

A volume of 1 µL of extracted RNA was employed to ascertain the concentration of the sample (ng/µL) through the utilisation of an ultra-micro spectrophotometer. The A260/A280 ratio was found to be between 1.8 and 2.1, indicative of high-quality RNA. The RNA-to-DNA reaction mixture was prepared in RNase-free 200 µL microcentrifuge tubes and thoroughly mixed to a total volume of 10 µL. The mixture was then incubated at 42°C for 2 min and subsequently maintained at 4°C. The cDNA synthesis reaction system was similarly prepared on ice in RNase-free 200 µL microcentrifuge tubes, mixed to a total volume of 20 µL. The reactions were conducted in accordance with the specified protocol using a gradient PCR instrument and subsequently stored at 4°C. The cDNA products were prepared individually in RNase-free microcentrifuge tubes, employing the following PCR reaction system, with three replicate wells per template, all configured on ice. A two-step PCR reaction programme was implemented.

Primers are as follows.

**Table:** 

Primer name	Primer sequence5′- 3′	PCR product length/bp
β-actin	CCTGGCACCCAGCACAAT	144
	GGGCCGGACTCGTCATAC	
C3	ACT​CAG​GCA​GTG​ACA​TGG​TG	270
	TGA​TGC​TCA​AGG​GCT​TCT​GG	
IRF7	ATG​GGC​AAG​TGC​AAG​GTG​TA	180
	GAT​GGT​ATA​GCG​TGG​GGA​GC	
Vcam1	AAT​GCC​TGG​GAA​GAT​GGT​CG	163
	AGG​AAA​AGA​GCC​TGT​GGT​GC	
CXCL10	AGC​TCT​ACT​GAG​GTG​CTA​TGT	85
	GTA​CCC​TTG​GAA​GAT​GGG​AAA​G	
AREG	CGC​TCT​TGA​TAC​TCG​GCT​CA	87
	CCC​CAG​AAA​ATG​GTT​CAC​GC	
Fnip2	GCA​TCA​TCC​CAA​GAA​GGC​TAT​GA	277
	CGC​AGT​CAG​TAA​GGC​AGC​AA	
ST6	TGA​GGT​CAC​GAT​CTG​GTG​GA	162
	TAC​AAG​ACG​CAC​AAC​CAG​CA	
Aldh1l2	ACC​AAG​AAA​GAG​CCA​CTC​GG	91
	CCA​AAC​ACG​CAG​CAC​TCT​TC	

### Extraction and identification of exosomes

Exosomes were isolated through a series of processes including centrifugation, column filtration, and purification. B7-H1^high^-MSCs and B7-H1^low^- MSCs supernatant was centrifuged at 2000 g for 30 min at 4°C. Then, the supernatant was transferred to a new centrifuge tube, and centrifuged at 10,000 × g for 45 min at 4°C to remove larger vesicles. Subsequently, the supernatant was filtered with a 0.45-μm filter membrane (Millipore, R6BA09493), and the filtrate was collected which was centrifuged again at 10,000 × g for 60 min at 4°C in a centrifuge. The supernatant was discarded, and the pellets were resuspended with 5 mL pre-cooled PBS. The pellets were centrifuged at 12,000 × g at 4°C for 2 min. The supernatant, which was rich in exosome particles, was retained. The harvested exosomes were transferred into the upper chamber of Exosome Purification Filter (Umibio) and centrifuged at 3,000 × g for 10 min at 4 °C. After centrifugation, the liquid at the bottom of the EPF column was collected, which was the purified exosomes. The isolated exosomes were subsequently stored at −80°C. Transmission electron microscopy (TEM) was employed to examine the morphological characteristics of the exosomes. Furthermore, the size and concentration of the exosomes were assessed utilizing a flow nanoanalyzer. The expression levels of CD9 and CD63 were determined via Western blot analysis.

### Scr and BUN measurements

The collected whole blood samples were subjected to centrifugation at 3,000 rpm for a duration of 15 min. Subsequently, Scr and BUN levels were quantified utilizing specific assay kits (Scr: RXWB0459-96, RUIXIN BIOTECH; BUN: RXWB0153-96, RUIXIN BIOTECH).

### Hematoxylin and eosin (H&E) staining

Kidney tissues were fixed in 4% paraformaldehyde for over 24 h and subsequently embedded in paraffin. The 5 μm thick sections were deparaffinised by immersion in xylene for 20 min, followed by rehydration through a graded series of anhydrous ethanol. H&E staining was conducted on the 5 μm-thick sections, which were deparaffinized using xylene and stained with H&E reagent (Sigma, United States). In brief, the sections were incubated with hematoxylin for 5 min at room temperature, rinsed, and then incubated with eosin for approximately 2 min at room temperature. The histomorphology of the kidney tissues was subsequently examined under a microscope.

### Western blot

Sample Preparation: Following the quantification of protein concentration, 20 μg of protein from each well was aliquoted (with an equivalent volume of culture supernatant serving as a control). The protein samples were then combined with 5× SDS-PAGE loading buffer, thoroughly mixed, and subjected to heat treatment at 95°C for 5 min. A 12% acrylamide separating gel was utilized, and the heated supernatant was loaded into each sample well for electrophoresis, conducted at 70 V for 30 min followed by 120 V for 60 min. Proteins were transferred onto a membrane using a constant current of 250 mA in a wet transfer system for 90 min. Closure: A 5% skimmed milk powder solution was sealed and maintained at room temperature for 1 hour. Incubation of the primary antibody involved diluting the antibody with 5% skimmed milk powder at a dilution ratio of 1:1000, followed by incubation at 4°C overnight. For the incubation of the secondary antibody, the primary antibody was aspirated, and the membrane was washed three times with TBST (5 min per wash). Subsequently, an HRP-labeled secondary antibody, diluted at a ratio of 1:5000, was added and incubated at room temperature for 1 h. For imaging development, following the secondary antibody incubation, the membrane was again washed three times with TBST (5 min per wash) and then developed using an ELC luminescent solution.

### ELISA

The concentrations of IL-1β (RX203063M, RUIXIN BIOTECH), IL-10 (RX203075M, RUIXIN BIOTECH), TNF-α (RX202412M, RUIXIN BIOTECH) and IL-18 (RX203064M, RUIXIN BIOTECH) in kidney tissues or macrophages were measured by enzyme-linked immunosorbent assay (ELISA) kits according to the manufacturer’s protocols. The OD values were read using a plate reader (Bio Rad).

### Whole transcriptome sequencing (RNA-seq)

The cDNA libraries were generated utilizing the NEBNext^®^ Ultra™ RNA Library Prep Kit for Illumina, provided by New England Biolabs, United States. Following this, the RNA quality was evaluated using an Agilent 2,100 Bioanalyzer from Agilent Technologies, United States. Sequencing of the samples was conducted on an Illumina HiSeq 6,000 system, manufactured by Illumina, United States. Differential expression genes (DEGs) had an average fold change of at least 2, and a q-value (FDR) less than 0.05. The sequences of.

### Statistical analysis

Statistical analyses were conducted utilizing R software (version 4.1.0). The assessment of normality was carried out via the Shapiro-Wilk test. Group comparisons were executed using the independent samples t-test for data exhibiting normal distribution and the Mann-Whitney U-test for data not conforming to normal distribution. For comparisons involving multiple groups, a one-way analysis of variance (ANOVA) was employed, supplemented by *post hoc* analyses using Tukey’s Honest Significant Difference (HSD) test where applicable. Categorical variables were analyzed through the chi-square test. A p-value of less than 0.05 was deemed indicative of statistical significance. All statistical analyses were conducted using two-tailed tests, and data visualization was performed utilizing GraphPad Prism software.

## Results

### Sorting of hucMSCs and expression of B7-H1 in positive and negative cells

**FIGURE 1:**
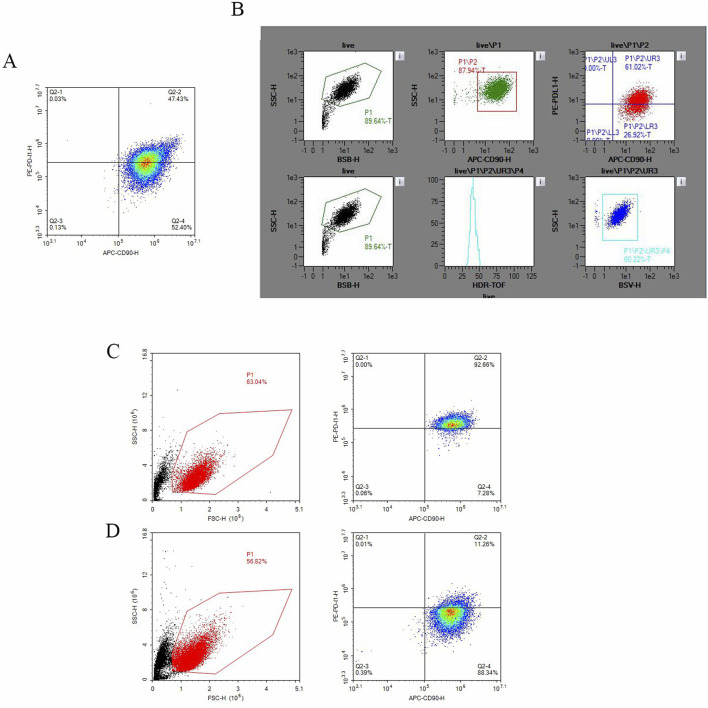
The sorting of hucMSCs and the expression of B7-H1 in both positive and negative cell populations. **(A)** Depicts the expression of B7-H1 in hucMSCs as determined by flow cytometry prior to sorting, **(B)** shows the flow cytometry sorting process for B7-H1^high^ and B7-H1^low^ hucMSCs, **(C)** the expression of B7-H1 in B7-H1^high^ hucMSCs as assessed by flow cytometry post-sorting and **(D)** the expression of B7-H1 in B7-H1^low^ hucMSCs as assessed by flow cytometry post-sorting.

Initially, we assessed the expression level of B7-H1 in hucMSCs via flow cytometry. The results indicated that the expression level of B7-H1 in hucMSCs was 47% (Figure 1A). Subsequently, we performed cell sorting on the hucMSCs (Figure 1B), resulting in the isolation of cell clusters with high and low B7-H1 expression, respectively. The post-sorting cell survival rates were 57% and 63%, respectively. In the low-expressing B7-H1 group, the expression of B7-H1 in cell clusters decreased from 47% to 11.26%, whereas in the high-expressing B7-H1 group, the proportion increased from 47% to 91.66% (Figures 1C,D).

### Identification of B7-H1^high^-Exo and B7-H1^low^-Exo

**FIGURE 2:**
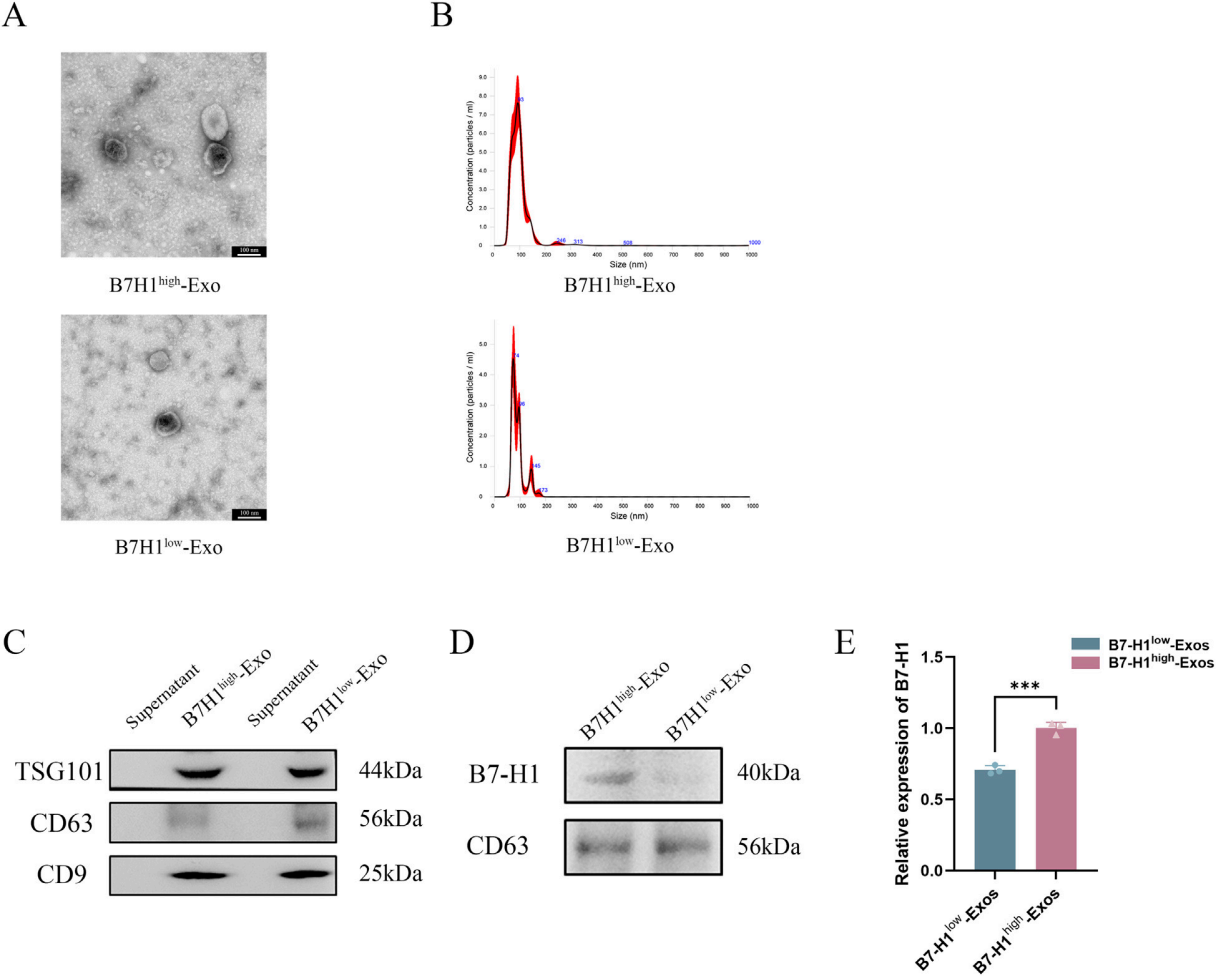
Characterization of B7-H1^high^-Exo and B7-H1^low^-Exo. **(A, B)** TEM and NTA were conducted to assess the size distribution of B7-H1^high^-Exo and B7-H1^low^-Exo. The scale bars 100 nm. **(C)** The expression levels of TSG101, CD63, and CD9 in the two groups. **(D)** The protein content of B7-H1 in B7-H1^high^-Exo and B7-H1^low^-Exo. **(E)** Quantification of the data presented in panel D was shown on the right. Data were expressed as mean ± standard error. Statistical significance was indicated as follows: ***P < 0.001 (n = 3).

B7-H1^high^-Exo and B7-H1^low^-Exo were characterized using TEM, NTA, and Western blotting. Under TEM, both groups of exosomes exhibited vesicle-like structures with a round or oval morphology (Figure 2A). NTA analysis further revealed that these exosomes had an average diameter of approximately 100 nm (Figure 2B). Western blot also confirmed that TSG101, CD9 and CD63 were positive in B7-H1^high^-Exo and B7-H1^low^-Exo (Figure 2C). Subsequently, Western blot analysis was conducted to assess the expression of B7-H1 in the two Exo groups. The results demonstrated that the B7-H1^high^-Exo group exhibited significantly elevated levels of B7-H1 protein compared to the B7-H1^low^-Exo group (Figure 2D). These findings indicated that B7-H1^high^-Exo was efficiently isolated from hucMSCs with high levels of B7-H1 expression, whereas B7-H1^low^-Exo was obtained from hucMSCs with lower expression levels of B7-H1.

### B7-H1^high^-Exo significantly enhances the restoration of renal function in mice subjected to renal ischemia-reperfusion injury

**FIGURE 3:**
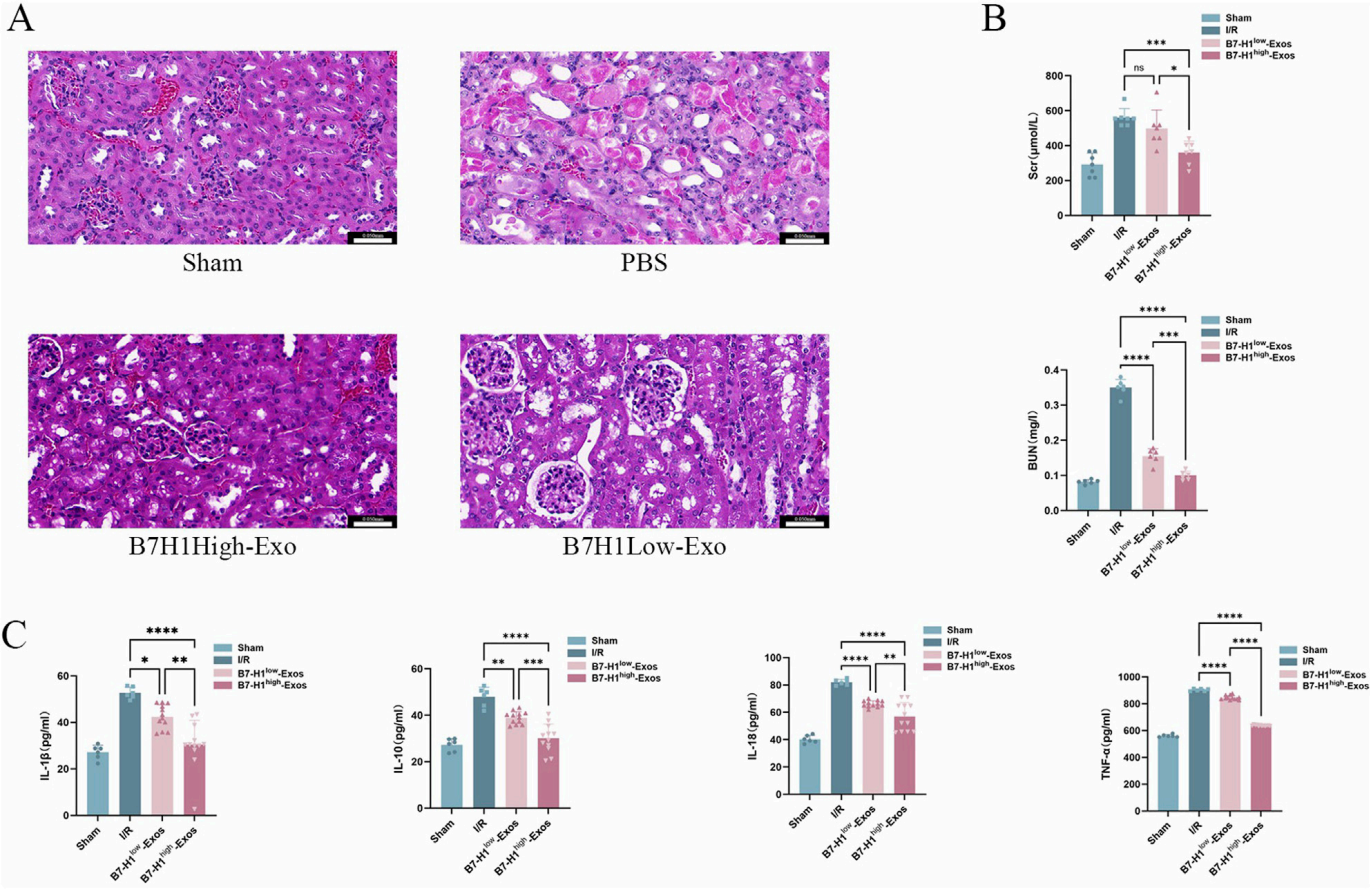
B7-H1^high^-Exo significantly enhanced the restoration of renal function in mice subjected to renal ischemia-reperfusion injury **(A)** Kidney tissues from four experimental groups were collected for histopathological analysis using H&E staining. Scale bars 0.050 mm **(B)** BUN and Scr levels were quantified using specific ELISA kits **(C)** The concentrations of interleukin-1β (IL-1β), interleukin-10 (IL-10), interleukin-18 (IL-18), and tumor necrosis factor-alpha (TNF-α) were measured with appropriate assay kits. Data are expressed as mean ± standard error. Statistical significance is indicated as follows: *P < 0.05, **P < 0.01, ***P < 0.001, ****P < 0.0001, ns denotes no significant difference (n = 5).

Our prior research demonstrated that exosomes derived from hucMSCs can be effectively targeted to the injured kidney, where they exerted significant therapeutic effects (Wei et al., 2024). In the present study, we employed a renal ischemia-reperfusion model to evaluate the impact of B7-H1^high^-Exo and B7-H1^low^-Exo treatments on BUN and Scr levels following renal IRI. The B7-H1^low^-Exo group exhibited a marked improvement compared to the Sham group (Figures 3A, B). Importantly, the B7-H1^high^-Exo group demonstrated superior functional recovery relative to the B7-H1^low^-Exo group. To further explore the therapeutic potential of Exo in IRI, we conducted histological analyses. Post-IRI, the extent of renal injury was markedly diminished in the group treated with B7-H1^low^-Exo compared to the IRI control group (Figures 3C, D). Remarkably, mice administered with B7-H1^high−^Exo demonstrated a reduced injury area compared to those receiving B7-H1^low^-Exo. Although the renal tubules remained sparsely packed, the tubular walls exhibited gradual homogenization, and the glomerular size progressively returned to normal in the B7-H1^low^-Exo group relative to the IRI group. In summary, our results indicated that kidneys treated with B7-H1^high^-Exo achieved superior functional recovery compared to those treated with B7-H1^low^-Exo.

### RNA-seq revealed multiple differentially expressed genes and pathways emerged in renal ischemia-reperfusion injury

**FIGURE 4:**
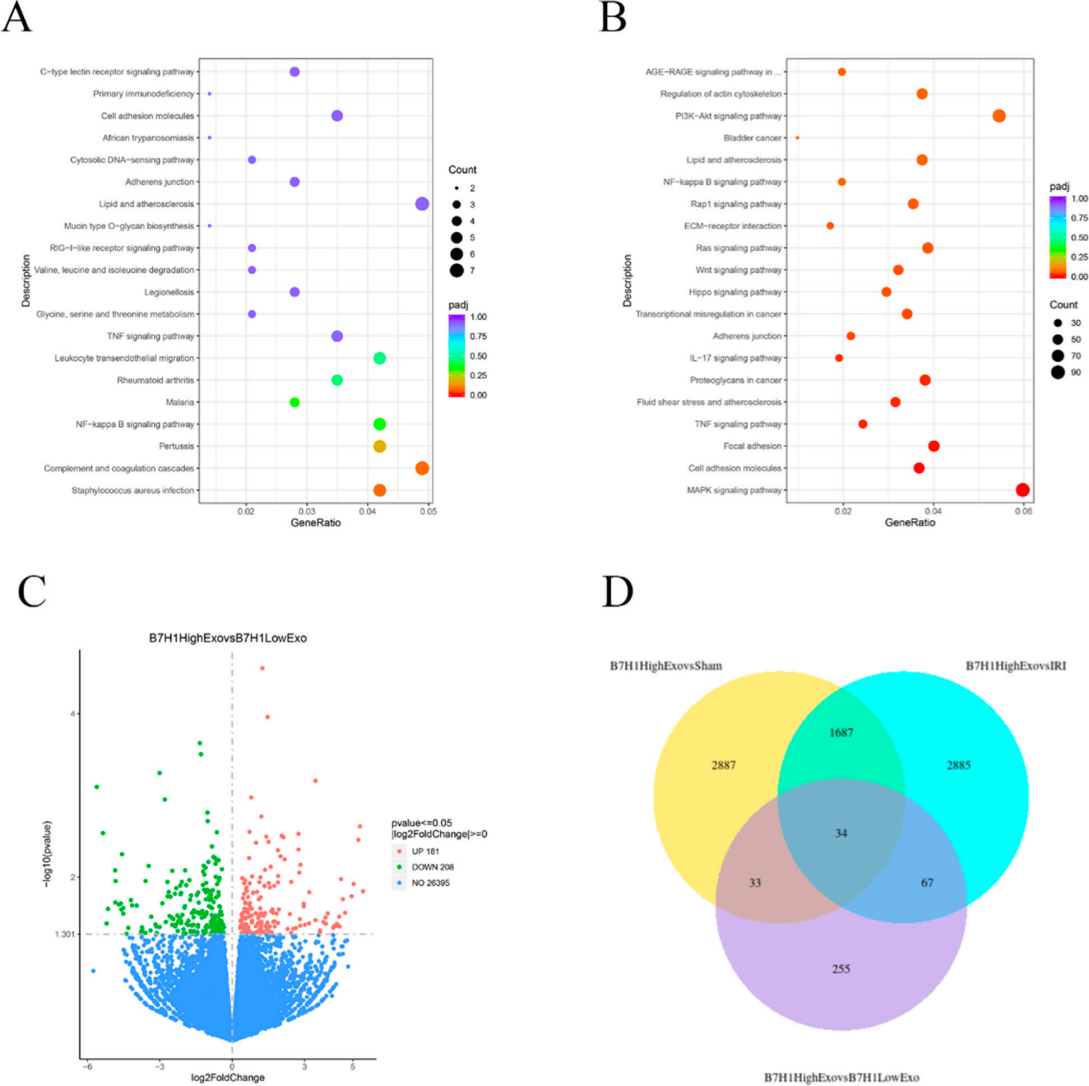
The effects of B7-H1^high^-Exo on renal ischemia-reperfusion injury-induced C3 expression. **(A)** Top 20 enriched KEGG pathways in the difference between B7-H1^high^-Exo and B7-H1^low^-Exo. **(B)** Top 20 enriched KEGG pathways in the difference between Sham and I/R. **(C)** Analysis of renal gene expression differences between the mouse B7-H1^high^-Exo and B7-H1^low^-Exo groups. **(D)** Venn of the four groups of differentially expressed genes.

To investigate the mechanisms underlying the B7-H1^high^-Exo-mediated repair of renal injury, we conducted a comparative analysis of the transcriptome profiles of renal tissues from mice in the four groups utilizing RNA-Seq. KEGG pathway enrichment analyses (Figures 4A, B) revealed that the differentially expressed genes between the B7-H1^high^-Exo and B7-H1^low^-Exo groups were predominantly enriched in pathways related to NF-κB signaling and TNF signaling pathway, among others. Meanwhile, we conducted a differential gene expression analysis on the B7-H1^high^-Exo and B7-H1^low^-Exo groups, revealing 181 upregulated and 208 downregulated genes (Figure 4C). Subsequently, we identified the top 10 most significantly upregulated and downregulated genes. Among these, eight genes—C3, IRF7, AREG, Cxcl10, Aldh1l2, Fnip2, Vcam1, and St6Galnac3—had been previously investigated in the context of ischemia-reperfusion injury. Venn can show the overlap of differential genes among different comparison combinations, and the differential genes that are common or unique to several comparison combinations can be screened by Venn diagram. Our analysis of differential genes in these four groups by Venn revealed a total of 34 overlapping differential genes (Figure 4D).

### B7-H1^high^-Exo mitigates the expression of C3 induced by renal ischemia-reperfusion injury

**FIGURE 5:**
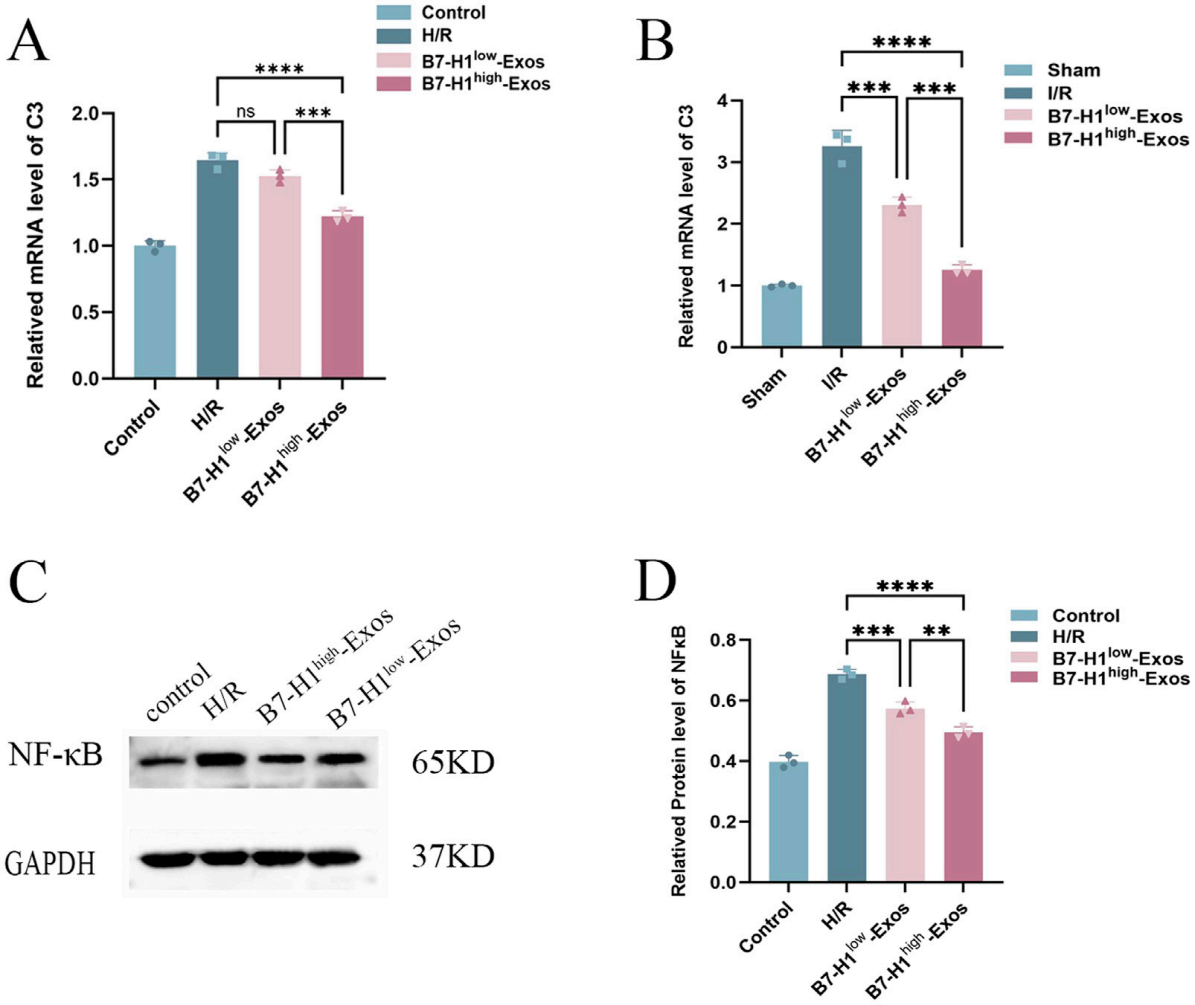
The effects of B7-H1^high^-Exo on renal ischemia-reperfusion injury-induced C3 expression, **(A, B)** the results of the RT-qPCR (n = 3). **(C, D)** Western blot analysis of NF-κB expression in normal HK-2 cells or hypoxia/reoxygenation-induced HK-2 cells with different treatments. The data were expressed as mean ± standard error. Statistical significance was indicated as follows: ***P < 0.001, ****P < 0.0001, ns denotes no significant difference.

To further ascertain which genes exhibited the most significant enhancement in functional recovery following IRI, we assessed their expression in renal IRI and hypoxia/reoxygenation-induced HK-2 cells using quantitative RT-qPCR. the expression level of the C3 protein was markedly reduced in HK-2 cells treated with B7-H1^high^-Exo compared to those treated with B7-H1^low^-Exo (Figure 5A). Surprisedly, consistent with the results of hypoxia/reoxygenation-induced HK-2 cells, expression of C3 was reduced in renal IRI (Figure 5B). The result suggested that B7-H1^high^-Exo might ameliorate renal IRI by downregulating C3 expression. A secondary validation of predictions from the RNA-seq analysis by RNAscope in Western blot confirmed a strong downregulation of NF-κB signaling (Figures 5C, D). This suggests that the downregulation of NF-κB is inseparable from the treatment of B7-H1^high^-Exo.

## Discussion

In this study, we have firstly identified a subpopulation of human umbilical cord MSCs characterized by high expression of B7-H1. We successfully isolated exosomes with elevated levels of B7-H1 from these cells. Our findings demonstrated that B7-H1^high^-Exo exhibit superior efficacy in repairing IRI. Through a series of experiments, we observed that B7-H1^high^-Exo facilitate the repair of renal tissues and the recovery of renal function by down-regulating complement C3. Similarly, B7-H1^high^-Exo compared to B7-H1^low^-Exo also showed repair of renal tissue by downregulation of NF-κB. These results strongly indicated the potential of B7-H1^high^ exosomes as a promising cell-free therapeutic approach for the treatment of IRI.

IRI is the primary contributor to AKI (Bonventre and Yang, 2011). Typically, the pathophysiological process of ischemia-reperfusion injury is delineated into six key components: vascular leakage, programmed cell death, transcriptional reprogramming, autoimmunity, activation of both innate and adaptive immune responses, and the no-reflow phenomenon (Eltzschig et al., 2011). Nevertheless, research focusing on the immune cell mechanisms underlying AKI remains scarce. During the initial phases of ischemia/reperfusion (I/R) injury, a substantial number of cells undergo necrosis as a result of hypoxic conditions. Necrotic cells, displaying potent immunostimulatory properties, promote the infiltration of inflammatory cells and cytokine production, leading to the subsequent release or upregulation of damage-associated molecular patterns (DAMPs), which are ligands associated with cellular injury or death (Hotchkiss et al., 2009). DAMPs interact with innate immune receptors, including toll-like receptors (TLRs), thereby activating the innate immune response (Chen et al., 2010; Iyer et al., 2009). In contrast to the well-documented immune response observed in I/R injury, the mechanisms by which the adaptive immune response is activated under sterile conditions remain inadequately understood. Existing research indicates that CD4^+^ and CD8^+^ T cells primarily mediate I/R injury (Day et al., 1950; Shen et al., 2009; Yilmaz et al., 2006). In contrast, recent studies suggest that regulatory T cells (Tregs) confer protection to the kidney against IR-induced inflammation and injury. However, the blockade of PD-1 on the surface of Tregs prior to graft transfer impairs their protective function against ischemic kidney injury. Additionally, the inhibition of B7-H1 or PD-L2 results in exacerbated kidney injury and inflammation (Jaworska et al., 2015). Consequently, B7-H1 presents significant potential for further investigation in the context of I/R injury.

MSCs can be obtained from a diverse array of sources, such as bone marrow, adipose tissue, umbilical cord, human placenta, dental pulp, skin, blood, and urine, in addition to induced pluripotent stem cells (iPSCs) (Huang and Yang, 2021). Clinical trials conducted thus far have demonstrated their substantial therapeutic potential (Abumoawad et al., 2019). Nevertheless, the variability in donor condition, cell type, differentiation capacity, and other influencing factors contributes to considerable heterogeneity, thereby constraining the effectiveness of MSC-based therapies (Kim et al., 2021). Growing evidence substantiates the role of Exo in stem cell-mediated repair through the modulation of immunomodulatory functions. In various models of kidney injury, Exo has been shown to facilitate the repair of damaged cells, promote the proliferation of renal tubular cells, and inhibit apoptosis and inflammation (Grange et al., 2019). Over time, MSC-Exo have gained prominence as a significant alternative therapeutic approach for diseases traditionally treated with MSCs. This is particularly evident in the pretreatment of stem cells, which allows for more precise disease management and minimizes treatment-related complications.

As previously noted, the heterogeneity among MSCs is regarded as a significant impediment to their clinical translation into therapies that are reproducible, predictable, and standardized (Zhou et al., 2021). This heterogeneity pertains to the variability in their molecular markers, differentiation potential, and biological functions. Such variation arises not only from diverse tissue sources but also from distinct cell subpopulations within the same tissue source (Hass et al., 2011; Mastri et al., 2014). While numerous studies have compared and analyzed the functional differences of MSCs derived from various tissue sources—encompassing aspects such as multidirectional differentiation potential and immunomodulatory functions—research focusing on the functional characteristics of MSC subpopulations within the same tissue remains limited (Baksh, 2007). Consequently, identifying subpopulations with specific functions and appropriately applying them could offer a viable strategy for tissue repair and functional restoration.

In this study, we identified a subpopulation of hucMSCs with the potential to enhance renal tissue repair and restore renal function using flow cytometry. However, it was observed that this subpopulation did not maintain stable expression during cell proliferation, eventually reverting to the expression profile of unsorted hucMSCs. So we isolated the subpopulation immediately after sorting and utilized them at passages 2–4 for both *in vivo* and *in vitro* experiments to solve the problem. The findings demonstrated that the *in vivo* administration of B7-H1^high^-Exo facilitated tissue restoration in regions affected by IRI and enhanced the recovery of renal function. Furthermore, *in vitro* experiments corroborated that the application of B7-H1^high^-Exo to HK-2 cells significantly attenuated apoptosis in renal tubular cells. Simultaneously, we conducted RNA-seq on the renal tissues of mice and identified differential expression in a total of 389 genes when comparing the B7-H1^high^-Exo and B7-H1^low^-Exo groups. Further validation using PCR *in vivo* and vitro revealed that the gene C3 demonstrated significant differential expression between the B7-H1^high^-Exo and B7-H1^low^-Exo groups. Notably, C3 is a critical complement protein situated at the convergence of all complement activation pathways. Extracellular, tissue, cell-derived, and intracellular C3 are pivotal in the dysregulated immune response observed in numerous diseases, rendering them promising therapeutic targets (Kolev et al., 2022). IRI represents an inevitable and severe consequence of renal transplantation, which significantly elevates the risk of delayed graft function and graft loss. The primary catalyst of detrimental response in the kidney is the activation of the complement system, a critical element of the innate immune system. The activation results in the deposition of complement C3 on renal tubules and the infiltration of immune cells, culminating in tubular damage and a consequent decline in renal function (Howard et al., 2021). In murine models, C3 deficiency confers a protective effect against renal IRI and diminishes immune cell infiltration (Zhou et al., 2000). Evidence suggests that following the induction of acute kidney injury in C3 knockout mice, the impairment of renal function is less pronounced compared to wild-type mice (Boudhabhay et al., 2020). Furthermore, Tregs are crucial in the context of IRI. Research demonstrated that the complement system also modulates the induction, function, and stability of Tregs (Van der Touw et al., 2013). A particular research group discovered that peripheral, murine, and natural regulatory T cells (nTregs) express the receptors C3aR and C5aR, which, through their signaling pathways, inhibit Tregs function (Kwan et al., 2013). CD4 Tregs are immunosuppressive T cells, and research has demonstrated that Tregs can mitigate AKI (Lee et al., 2010). Jaworska K et al. reported an improvement in IRI following Tregs transplantation, an effect contingent upon Tregs expression of programmed death ligands 1 and 2, i.e., B7-H1 and PD-L2. Concurrently, experimental evidence indicating PD-1 expression by renal tubular epithelial cells (Jaworska et al., 2015), along with clinical observations of renal adverse events in patients undergoing treatment with immune checkpoint inhibitors targeting the PD-1/B7-H1 axis (Wanchoo et al., 2017), underscores the significance of PD-1 in renal inflammation. Consequently, we hypothesize that B7-H1^high^-Exo may enhance the function of Tregs by down-regulating C3 and the associated complement cascade pathway, thereby inhibiting renal inflammation. This hypothesis warrants validation in future studies.

However, this study is not without limitations. Firstly, although we have verified how B7-H1^high^-Exo acts on C3 and NF-κB to repair damage separately, the interaction mechanisms among B7-H1, C3, and NF-κB required further elucidation. Furthermore, the *in vivo* administration of exosomes through the tail vein did not constitute a non-invasive or direct method of drug delivery. Future research could explore transnasal drug delivery, which is currently favored as the preferred method for exosome-based drug delivery due to its numerous advantages. Initially, it offers a non-invasive and direct approach to drug delivery, complemented by its rapid therapeutic effects (Long et al., 2017). Ultimately, this study concentrated on the mechanisms associated with renal tissue repair and functional recovery following IRI.

## Conclusion

In summary, our study represented the inaugural investigation to elucidate the roles of C3 and NF-κB through B7-H1^high^-Exo in the context of IRI, unveiling their potential as therapeutic targets in this pathological setting. The reparative capacity of hucMSCs-derived exosomes with high B7-H1 expression was significantly greater than that of exosomes with low B7-H1 expression. Furthermore, these exosomes facilitated renal tissue repair and functional recovery by down-regulating C3 and NF-κB. Therefore, the therapeutic strategy centered on B7-H1 might effectively augment the ameliorative impact of hucMSCs-derived exosomes on renal injury resulting from IRI.

## Data Availability

The original contributions presented in the study are publicly available. This data can be found here: https://pan.baidu.com/s/1CYgX-egq6MiOHx5CeDT_-w?pwd=ryf6 Access code: ryf6.
